# Mitochondrial and inflammatory changes in sporadic inclusion body myositis

**DOI:** 10.1111/nan.12149

**Published:** 2015-03-04

**Authors:** Karolina A. Rygiel, James Miller, John P. Grady, Mariana C. Rocha, Robert W. Taylor, Doug M. Turnbull

**Affiliations:** ^1^ Wellcome Trust Centre for Mitochondrial ResearchInstitute for Ageing and HealthThe Medical SchoolNewcastle UniversityNewcastle upon TyneUK; ^2^ Newcastle University Centre for Brain Ageing and VitalityInstitute for Ageing and HealthThe Medical SchoolNewcastle UniversityNewcastle upon TyneUK; ^3^ Department of NeurologyNewcastle upon Tyne Hospitals NHS Foundation Trust Royal Victoria InfirmaryNewcastle upon TyneUK

**Keywords:** atrophy, complex I, inflammation, mitochondria, mitochondrial deletions, sIBM

## Abstract

**Aims:**

Sporadic inclusion body myositis (sIBM) is the most common late onset muscle disease causing progressive weakness. In light of the lack of effective treatment, we investigated potential causes underlying muscle wasting. We hypothesized that accumulation of mitochondrial respiratory deficiency in muscle fibres may lead to fibre atrophy and degeneration, contributing to muscle mass reduction.

**Methods:**

Histochemical and immunohistochemical analyses were performed on muscle biopsies from 16 sIBM patients to detect activity of mitochondrial enzymes and expression of mitochondrial respiratory chain proteins along with inflammatory markers respectively. Mitochondrial DNA mutations were assessed in single muscle fibres using real‐time PCR.

**Results:**

We identified respiratory‐deficient fibres at different stages of mitochondrial dysfunction, with downregulated expression of complex I of mitochondrial respiratory chain being the initial feature. We detected mitochondrial DNA rearrangements in the majority of individual respiratory‐deficient muscle fibres. There was a strong correlation between number of T lymphocytes and macrophages residing in muscle tissue and the abundance of respiratory‐deficient fibres. Moreover, we found that respiratory‐deficient muscle fibres were more likely to be atrophic compared with respiratory‐normal counterparts.

**Conclusions:**

Our findings suggest that mitochondrial dysfunction has a role in sIBM progression. A strong correlation between the severity of inflammation, degree of mitochondrial changes and atrophy implicated existence of a mechanistic link between these three parameters. We propose a role for inflammatory cells in the initiation of mitochondrial DNA damage, which when accumulated, causes respiratory dysfunction, fibre atrophy and ultimately degeneration of muscle fibres.

## Introduction

Sporadic inclusion body myositis (sIBM) is the most common myopathy of older age, with onset typically after the age of 50 years [1]. sIBM is categorized as one of the idiopathic inflammatory myopathies along with dermatomyositis, polymyositis and necrotizing autoimmune myositis [2]. However, in contrast to these other disorders, there is no established role for immunosuppressive treatment in the management of patients with sIBM. This is likely to reflect the contribution of degenerative mechanisms to the pathogenesis in addition to an abnormal immune response.

In sIBM, there is a distinctive pattern of muscle involvement which forms a core element of the clinical diagnosis [3]. Muscle biopsy reveals a combination of excessive inflammation (consisting mostly of CD8‐positive T lymphocytes) [4] with widespread upregulation of sarcolemmal and sarcoplasmic major histocompatibility complex‐I (MHC I) with degenerative features that include abnormal sarcoplasmic protein aggregation and increased mitochondrial impairment [5, 6, 7].

Mitochondrial abnormalities are very common in muscles of sIBM patients and they include diminished respiratory chain function, abnormal proliferation and accumulation of mitochondria [6]. In Oldfors and colleagues' initial study of muscle biopsies from three sIBM patients, there was low or absent cytochrome *c* oxidase (COX) activity, despite normal succinate dehydrogenase (SDH) activity, in 2–5% of all muscle fibres (deltoid muscle). Less than 1% of COX‐deficient fibres were observed in age matched controls, indicating that the mitochondrial histochemical changes in sIBM were related to disease pathology and not just a consequence of normal ageing. *In situ* hybridization analysis of tissue sections provided evidence for the existence of mitochondrial DNA (mtDNA) deletions, which was further confirmed with Southern blotting [6]. An extended study of 20 additional sIBM patients demonstrated the frequency of respiratory chain deficiency to affect 0.5–5.0% of all fibres [8]. The potential importance of the mitochondrial changes in sIBM is highlighted by the fact that COX‐deficient fibres are the most consistent abnormal histopathological feature in muscle biopsies from sIBM patients after MHC I upregulation, present in 98% of cases compared with 90% with rimmed vacuoles or inclusions [9]. Molecular analysis of a number of individual fibres revealed the presence of different large‐scale mtDNA deletions and only one point mutation, in a tRNA gene. It is still unknown why mtDNA mutations accumulate in sIBM and what the consequence of the respiratory deficiency is to the disease progression.

The aetiology of these changes in the muscle of patients with sIBM is still unclear but there is growing evidence that both inflammation and degeneration can play a role. Findings from another autoimmune disease, multiple sclerosis, showed abundant population of respiratory‐deficient neurones in patients' brains [10]. Mitochondrial injury in this disease may be triggered by reactive oxygen (ROS) and nitric oxide species (RONS) generated by activated microglia and macrophages, which are frequently found within multiple sclerosis lesions [11].

In this study we wished to examine the prevalence of respiratory deficiency and its relationship to the inflammation and atrophy in muscle biopsies from patients with sIBM. We have investigated the cellular phenotype in muscle tissue from 16 sIBM patients. We were interested to establish whether the rate of the accumulation of the mitochondrial defect in muscle tissue relates directly to the clinical phenotype observed in these patients as well as determining whether the severity of mitochondrial abnormalities, atrophy and inflammation could be indicative of disease progression.

## Methods

### Patients and muscle biopsies

Open muscle biopsies obtained from 16 sIBM patients were used in this study. All patients included in the study had a diagnosis of clinically defined or pathologically defined sIBM according to recent criteria [3]. Disease progression was estimated in 10 of 16 patients using serial hand‐held myometry (Citec) over a mean of 3.5 years (range 2–7 years). Strength in Newtons was determined for muscle groups in upper and lower limbs. Muscle groups assessed included shoulder abduction, elbow flexion and extension, wrist extension, pinch and grip, hip flexion, hip abduction, knee extension and dorsiflexion. The selection of muscle groups varied among patients with a mean of seven groups assessed (range three to eight) either unilaterally on the weaker side [4] or bilaterally [6]. Knee extension and grip strength were both determined in all but one patient. Measurements were performed in standardized fashion by a trained physiotherapist. For each muscle group, at least two measurements were performed to obtain values within 10% variation and the mean of these two determined. Change in strength was calculated as a percentage relative to baseline.

All muscle biopsies were taken from *vastus lateralis*, except for two biopsies which were taken from *deltoid* and *gastrocnemius*, frozen in isopentane immersed in liquid nitrogen and stored at −80°C. Clinical information about the patients is summarized (Table S1). Ethical approval was granted and consent for research was obtained from all individuals taking part in the study.

### Histological and immunohistochemical analyses

Frozen biopsies were cut at 10 μm and selected sections were used for haematoxylin and eosin (H&E) staining according to an established protocol. Other sections were used for sequential cytochrome *c* oxidase and succinate dehydrogenase (COX/SDH) histochemistry in order to detect respiratory‐deficient fibres as previously described [12]. Sections used for molecular analysis were cut at 20 μm, mounted onto polyethylenenaphthalate (PEN) slides (Leica Microsystems, Milton Keynes, UK) and reacted for SDH activity only, as the 3,3′‐Diaminobenzidine (DAB) used in COX reaction solution interferes with some downstream mtDNA analyses [13].

Serial sections were cut at 10 μm and dried at room temperature for 1 h. The sections were fixed with 4% PFA (Sigma, Gillingham, Dorset, UK) permeabilized in a gradient of methanol and endogenous peroxidase activity was blocked by incubation with 0.3% hydrogen peroxide. Nonspecific protein interactions were quenched by incubation with the blocking solution [5% bovine serum albumin/Tris‐buffered saline and Tween 20 (BSA/TBST)]. Primary antibodies recognizing mitochondrial proteins (outer mitochondrial membrane protein: anti‐porin, matrix protein: anti‐PDH E2, complex I subunits: anti‐cI‐19 and anti‐cI‐20, complex IV subunits: anti‐COX‐I and anti‐COX‐IV, complex II subunit: cII‐70) and inflammatory cells (lymphocytes: anti‐CD3 and macrophages: anti‐CD68) were diluted to optimal concentrations in the blocking solution (Table S2). The antibodies were applied onto the section for an overnight incubation at +4°C. All of the antibodies detecting mitochondrial proteins were purchased from Abcam (Cambridge, UK) and both CD3 and CD68 antibodies were acquired from DAKO (Ely, UK). The following day, slides were washed in TBST and the tissue sections incubated with mitochondrial complexes antibodies were then incubated with the universal probe followed by polymer‐horseradish peroxidase (HRP) according to manufacturer's instructions (X‐Cell plus Polymer HRP detection kit, MenaPath, A. Menarini Diagnostics). Sections which had been immune‐reacted with anti‐CD3 and anti‐CD68 antibodies were incubated with secondary anti‐mouse biotinylated antibody (Vector Laboratories, California, USA) for 1.5 h at room temperature, then washed and incubated with ABC peroxidase complex for 30 min (Vector Laboratories). Signal was developed with DAB chromogen solution (SigmaFast tablets, Sigma) and the sections were counter‐stained with Mayer's Haematoxylin. Slides were dehydrated, cleared in Histo‐Clear II (National Diagnostics, Atlanta, GA, USA) and mounted in DPX mounting medium (Sigma). Images of serial sections were obtained using Zeiss Axio Vision microscope (Carl Zeiss, Oberkochen, Germany).

### Serial tissue sections – experimental set‐up

**Figure 1:**
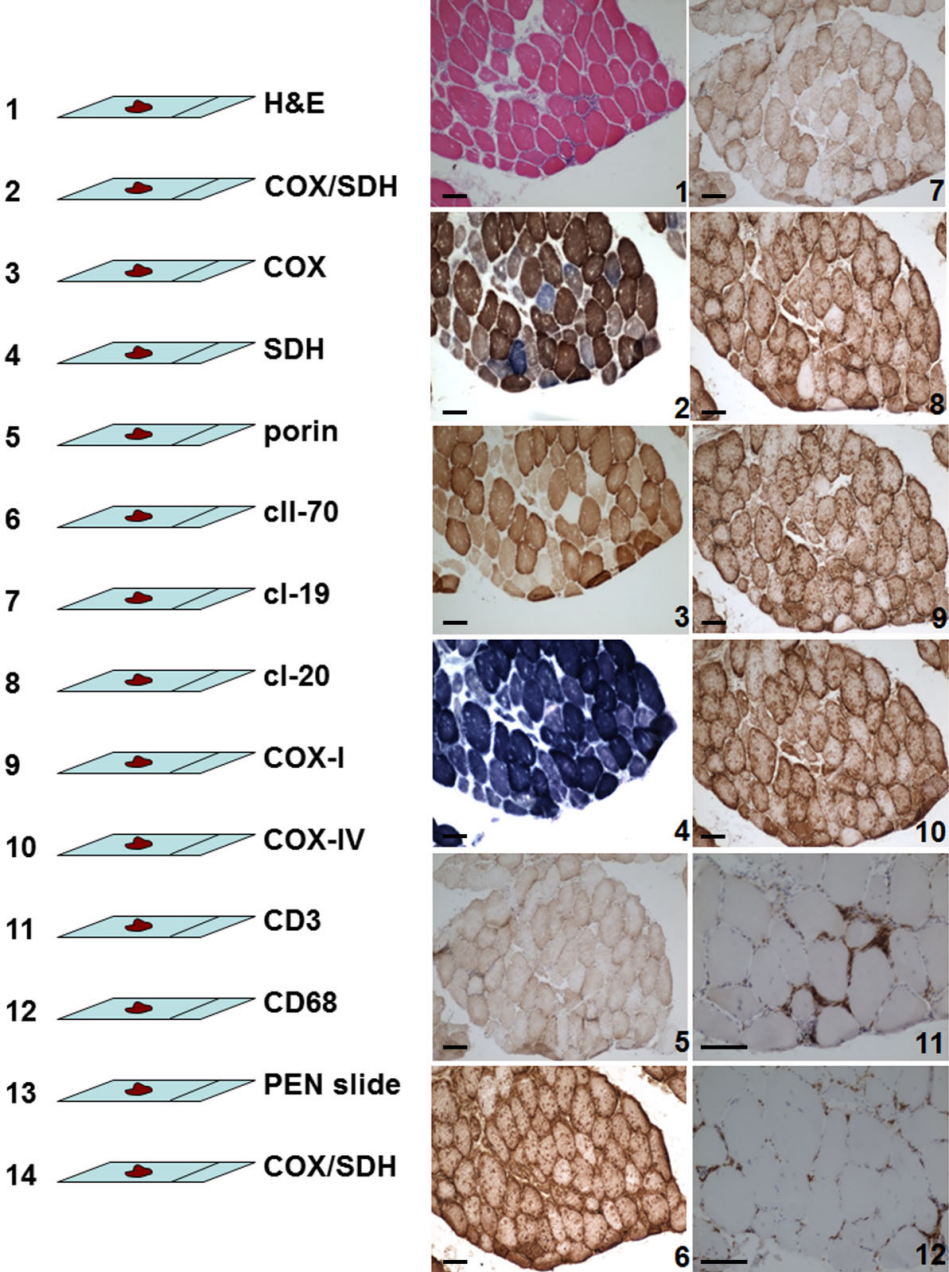
Overview of the experimental set‐up for the assessment of serial muscle sections from inclusion body myositis muscle biopsies. The scale bars measure 100 μm.

Serial sections were stained in a fixed order to ensure robust and comparable analyses between the patients (Figure 1). Individual muscle fibres were traced in all sections and their characteristics reported. Sequential COX/SDH histochemistry was undertaken on the first and last section of the series to verify whether an individual muscle fibre had the same biochemical phenotype throughout the analysed length (Figure 1). Complete analysis was only possible on 13 biopsies because in the remaining three biopsies one or more of sections from a series failed to produce a reliable antibody labelling or the muscle morphology was affected by freezing or storage procedure. It is worth noting that although patient 11 and 13 had biopsies taken from deltoid or gastrocnemius respectively rather than quadriceps muscle (Table S1), neither of them presented unusual results following histochemical and immunohistochemical analyses.

### Real‐time PCR analysis of DNA extracted from single cells

Frozen muscle sections (20 μm), mounted on PEN slides and reacted for SDH activity, were used for mtDNA analyses. Single muscle fibres of interest were dissected out using Leica Laser Microdissection System (AS‐LMD) or PALM MicroBeam (Carl Zeiss). The cells were then lysed as previously described [14]. Analysis of deletion levels in mtDNA were carried out using multiplex real‐time PCR assay to detect two mitochondrial genes, *MT‐ND1* and *MT‐ND4* according to a previously optimized protocol [15, 16].

### Whole mitochondrial genome cycle sequencing

The method to determine the sequence of the entire mitochondrial has been described elsewhere [17]. This was determined in five individual muscle fibres from one patient (patient 7). Sequencing results were compared with the revised Cambridge sequence [18, 19].

### Counting of fibres and inflammatory cells

The proportion of respiratory‐deficient cells (COX‐deficient) and complex I subunit 19‐deficient cells (cI‐19‐deficient) was presented as a percentage of a total number of fibres counted (*n* = 150 ± 15). Atrophy level was assessed by counting approximately 500 (*n* = 500 ± 55) fibres in each muscle biopsy. Only fibres with the smallest diameter of less than 30 μm were considered atrophic and were included in calculations. A larger number of fibres were counted for the muscle atrophy analysis than the COX/SDH analysis given COX‐deficient fibres were more frequent and distributed in a mosaic pattern whereas atrophic fibres less common and sometimes unevenly distributed. For both the COX deficiency and atrophy counts, randomly selected areas were chosen for assessment, which was carried out using a stereological system (Stereo Investigator, MBF Bioscience, Williston, VT, USA). Inflammatory cells were rarely evenly distributed within the muscle tissue therefore in order to quantify abundance of T lymphocytes (CD3‐positive) and macrophages (CD68‐positive) a representative area was selected for each case. The representative area (0.7–2.5 mm^2^) reflected inflammatory status of the entire muscle section. Results were presented as a number of either T cells or macrophages per mm^2^ of tissue.

Each of these counts was carried out independently with no knowledge of the previous results. The counts were verified by an independent investigator in three randomly chosen cases and the difference in results obtained by the two independent investigators did not exceed 10%. This discrepancy may have been caused by a different region of the biopsy analysed by the second investigator.

### Statistical analysis

Statistical analyses of the data were carried out using MiniTab 16. Normally distributed data were analysed with *T*‐tests and nonparametric data with appropriate nonparametric tests including Mann–Whitney and Wilcoxon signed rank test. The difference between data sets was considered statistically significant only when *P* was equal or lower than 0.05. When data were analysed using multiple tests *P* was adjusted accordingly.

## Results

### Muscle fibres containing mitochondrial abnormalities are common in sIBM patients

**Figure 2:**
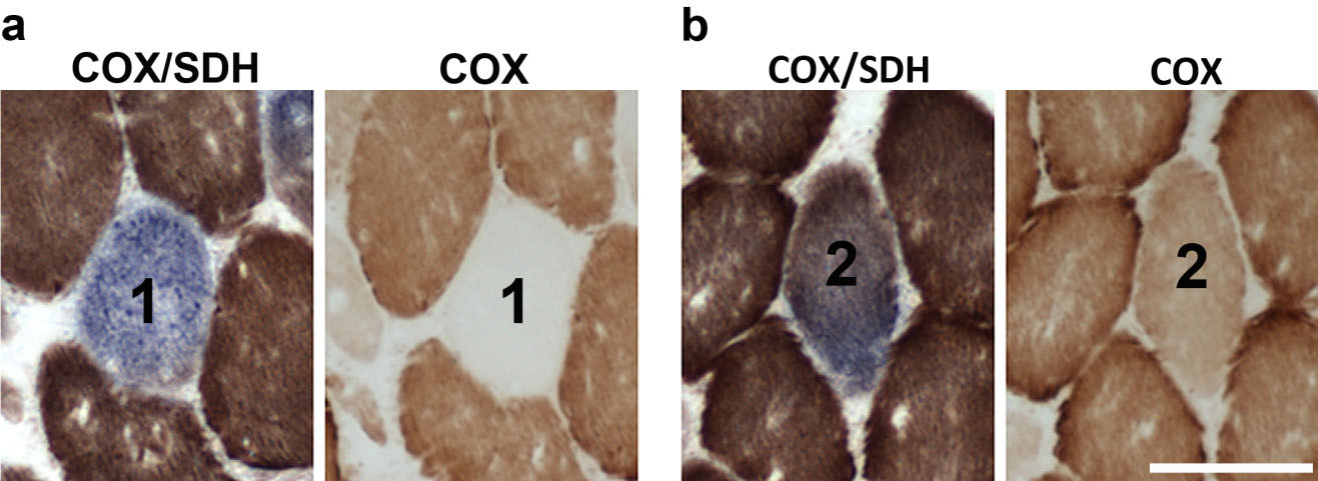
The demonstration of muscle fibres with impaired mitochondrial respiration in sIBM biopsies demonstrated by combined COX/SDH histochemistry and COX histochemistry alone. The muscle biopsy sections shown are from patient 10. (**a**) Entirely COX‐deficient fibres (no COX activity detected with COX histochemistry alone; fibre 1) and (**b**) intermediate fibres (reduced COX activity; fibre 2) were found in every sIBM biopsy. Scale bar is 100 μm for all images.

**Figure 3:**
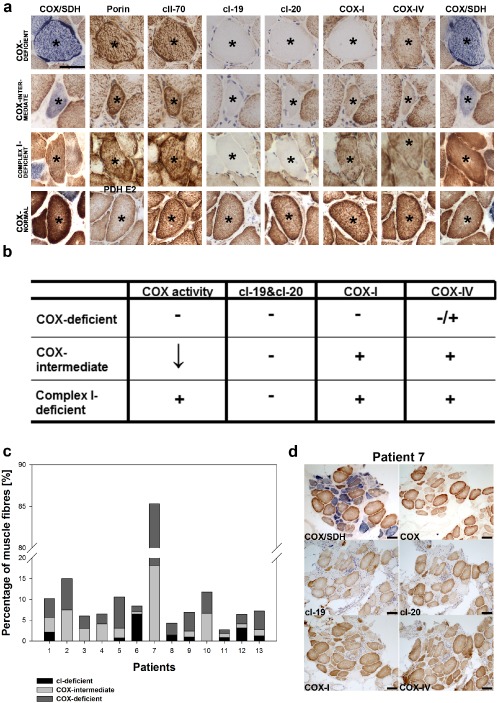
Characterization of the mitochondrial respiratory phenotype in muscle fibres from sIBM muscle biopsies. (**a**) Selected components of mitochondrial respiratory chain were detected by immunohistochemistry (IHC) and COX/SDH histochemistry. Analyses were performed on transverse serial sections (asterisks depict a single muscle fibre in sequential cryosections). Four categories of fibres were identified: fully respiratory‐deficient (COX‐deficient), partially respiratory‐deficient (COX‐intermediate), lacking complex I expression (cI‐deficient) and respiratory normal (COX‐normal). COX/SDH histochemistry was carried out on the first and the last of serial sections to confirm the same phenotype. Porin or PDH E2 (dihydrolipoamide transacetylase of pyruvate dehydrogenase) were used as indicators of mitochondrial mass. Images were acquired at 20× magnification, the scale bar displayed in the first image measures 50 μm and applies to all images in this panel. (**b**) Summary of phenotype of three groups of abnormal fibres in sIBM. ‘−’ signifies lack of enzyme activity or protein expression, ‘+’ normal activity of expression, ‘+/−’ presence or absence of protein, reduced enzymatic activity. (**c**) Percentage of each of the three groups of pathological fibres in biopsies from 13 sIBM patients. (**d**) Histochemistry and IHC carried out on a biopsy from patient 7 who showed the highest deficiency levels in COX activity and components of mitochondrial complexes. The scale bars measure 100 μm.

In agreement with reports from other groups [6], we confirmed presence of muscle fibres with evidence of mitochondrial dysfunction in *vastus lateralis*, *deltoid* or *gastrocnemius* biopsies obtained from 16 sIBM patients (Table S1). Using COX/SDH histochemistry we distinguished two populations of fibres bearing mitochondrial defects – cells without any detectable COX activity (COX‐deficient fibres) (Figure 2 **a**) and cells with markedly reduced levels of this enzyme (COX‐intermediate fibres) (Figure 2 **b**) [13]. Subsequently, we labelled serial muscle biopsy sections with a range of antibodies detecting different protein components of the mitochondrial respiratory chain (Figure 3 **a**). Cells entirely lacking COX activity expressed neither complex I subunits: cI‐19 and cI‐20 nor mitochondrially encoded subunit I of complex IV (COX‐I) and had either preserved or reduced expression of nuclear encoded subunit IV of complex IV (COX‐IV). Similarly, we failed to detect expression of either of the two subunits of complex I in COX‐intermediate fibres. These fibres, however, had preserved expression of COX‐I. Most intriguingly, we distinguished a group of fibres with normal COX activity and complex IV protein level but entirely abolished expression of complex I subunits. These data lead to formulation of three categories of abnormal muscle fibres found in sIBM biopsies: COX‐deficient, COX‐intermediate and complex I‐deficient (Figure 3 **b**). All three groups of aforementioned fibres showed high levels of both complex II and porin expression implicating that reported changes in expression of complex I and IV were not a consequence of a low mitochondrial density within the cells. Proportion of each of the three groups of abnormal muscle fibres in individual biopsies from sIBM patients is presented (Figure 3 **c**). The biopsy from patient 7, which demonstrated a particularly high level of COX‐deficient and COX‐intermediate fibres, is also presented (Figure 3 **d**).

**Figure 4:**
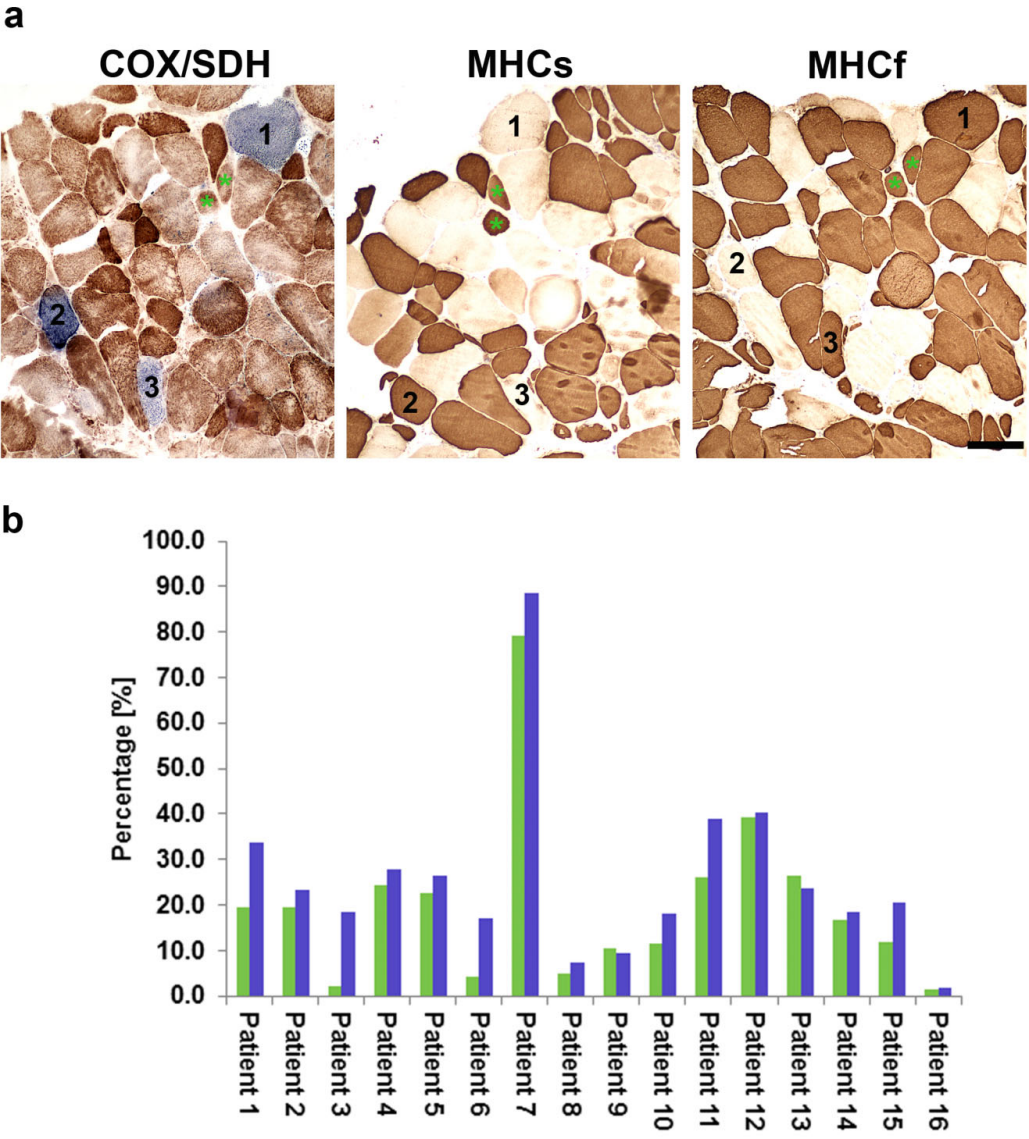
Immunohistochemistry for myosin heavy chain slow and fast (MHCs and MHCf respectively) and contribution of respiratory‐deficient and complex I‐deficient fibres to the total number of fibres in biopsies. (**a**) Fibres depicted with numbers demonstrate that both slow and fast twitch fibres are affected by mitochondrial deficiency. There are some fibres that co‐express both MHC isoforms (marked with green asterisks). (**b**) Percentage of COX‐deficient (green bars) and complex I‐deficient (blue bars) of the entire fibre population. On average 150 fibres were counted in each biopsy, 16 sIBM cases were analysed. Group of muscle fibres with deficient complex I (complex I‐deficient) was statistically more abundant than COX‐deficient (Wilcoxon signed rank test, *P* = 0.002). The scale bar corresponds to 100 μm and applies to all images.

Respiratory deficiency was not restricted to one fibre type. Both slow (Type I) and fast (Type II) twitch fibres encompassed a portion of fibres with defective oxidative phosphorylation as assessed on the basis of COX inactivity (Figure 4 **a**).

### Investigating the degree of respiratory deficiency in muscle biopsies from patients with sIBM

Quantitative analysis of COX/SDH histochemistry revealed that the combined proportion of COX‐deficient and COX‐intermediate fibres varied between 1.5% and 79.1% of affected fibres. Complex I‐deficient fibres constituted between 1.7% and 88.7% of total fibres counted and, more importantly, in the great majority of biopsies complex I‐deficient fibres were more abundant than COX‐deficient cells (Figure 4 **b**). Statistical analysis of all cases showed that complex I‐deficient fibres were more abundant than COX‐deficient fibres in the analysed cohort of patients (Wilcoxon signed rank test, *P* = 0.002). Levels of neither COX deficiency nor complex I deficiency correlated with the age of disease onset (*r* = −0.39, *P* = 0.02704 and *r* = −0.48, *P* = 0.1611 respectively). Additionally, strength decline, measured by myometry, failed to correlate with either COX deficiency (*r* = 0.11, *P* = 0.7614) or complex I deficiency (*r* = 0.24, *P* = 0.5020).

### Respiratory deficiency is caused by mtDNA deletions in a proportion of abnormal muscle fibres

**Figure 5:**
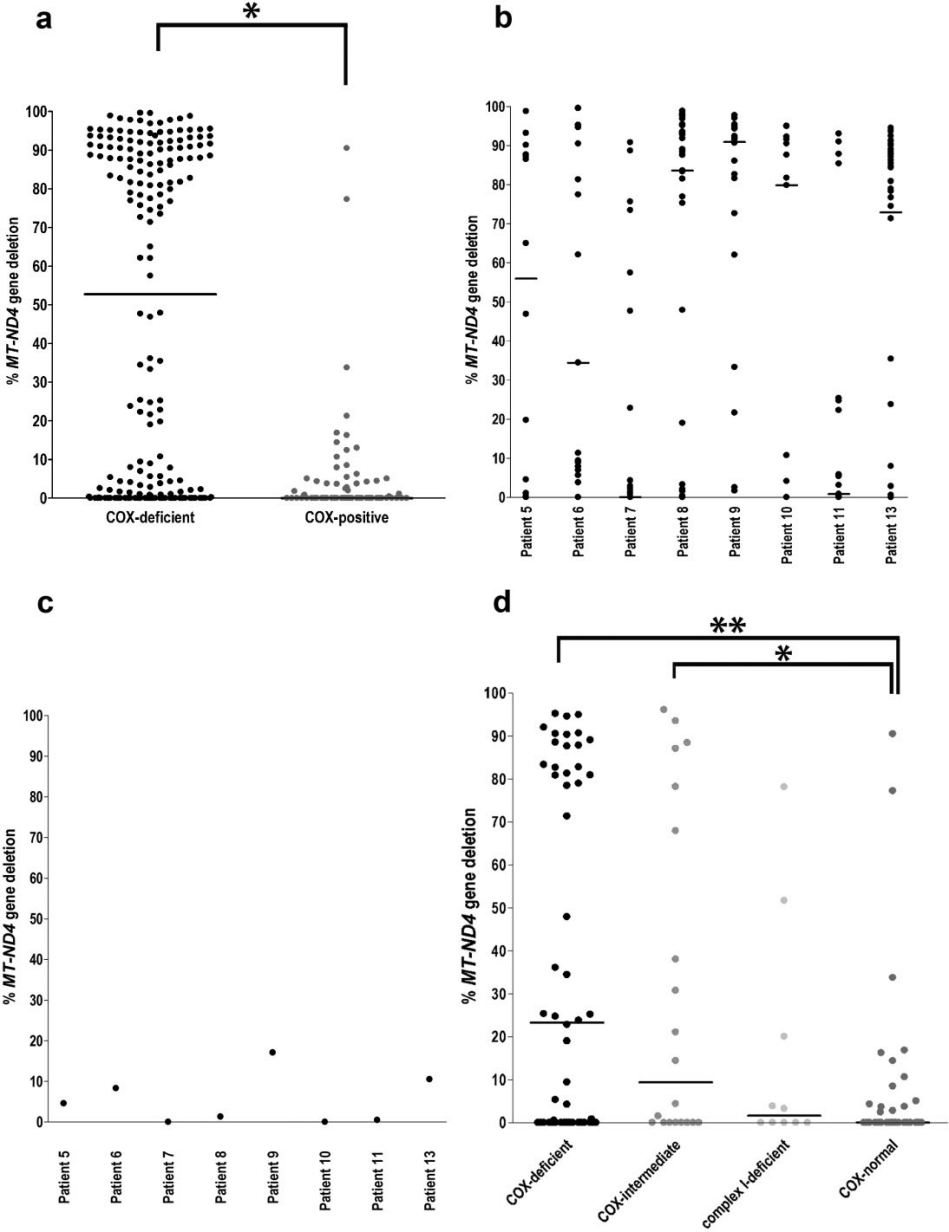
mtDNA deletion load detected in single muscle fibres as well as muscle homogenates from eight sIBM patients. (**a**) Combined single fibre mtDNA deletion data from all eight cases were plotted to demonstrate difference between the entire COX deficient (190 cells) and COX normal (62 cells) muscle fibre population. Horizontal lines indicate the median. The difference in deletion load between COX‐deficient and COX‐normal cells was statistically significant (Mann–Whitney test, *P* < 0.0001). Each dot signifies a reading from one cell. (**b**) mtDNA deletion levels from individual respiratory‐deficient cells were plotted for each patient to indicate variability between patients. (**c**) mtDNA deletion load in muscle homogenate DNA from the same eight sIBM patients. (**d**) individual muscle fibres of one of the four categories: COX‐deficient (54 cells), COX‐intermediate (20 cells), complex I‐deficient (10 cells) and COX normal (40 cells). The cells were characterized based on histochemistry for COX/SDH activity and IHC for selected mitochondrial respiratory chain components. Horizontal lines indicate the median. Statistically significant difference was only achieved for COX‐deficient *vs.* COX‐normal (**P = 0.0003) and COX‐intermediate and *vs.* COX‐normal groups (*P = 0.0135) (Mann–Whitney test).

We used multiplex real‐time PCR assay to investigate whether fibres expressing a respiratory‐deficient phenotype carried high levels of mtDNA deletions as previously reported [8]. Analysis was carried out on DNA extracted from single muscle fibres from eight patients and data were collected from 190 COX‐deficient and 62 COX‐normal fibres. As expected, the overwhelming majority (95%) of COX‐normal cells contained a very low level of mtDNA deletion (Figure 5 **a**). COX‐deficient cells could be grouped into three categories based on their deletion content. Muscle fibres with a ‘high deletion load’ (60–99%) were the most abundant group and comprised 51% of respiratory‐deficient fibres, ‘medium deletion load’ group (30–59%), encompassed only 4% of cells and ‘no deletion’ group was represented by 45% fibres (Figure 5 **a**). The difference in *MT‐ND4* deletion load between COX‐normal and COX‐deficient group was highly statistically significant (Figure 5 **a**, Mann–Whitney test, *P* < 0.0001).

We noted high level of variability between individual patients. We measured mtDNA deletion levels in eight individual cases in between 14 and 45 COX‐deficient muscle fibres per case (Figure 5 **b**). Most patients showed a wide distribution in mtDNA deletion load in individual fibres from the COX‐deficient group. Patient 7 is an outlier and the particularly high level of COX deficiency in this patient's muscle is currently difficult to explain. Majority of single cells as well as the homogenate analysis have not revealed mtDNA deletion using our *MT‐ND1*/*MT‐ND4* assay. We have also sequenced the entire mitochondrial genome from five COX‐deficient cells from this patient but did not detect any pathogenic mtDNA point mutations. Multiple mtDNA deletions were clearly detected by long range PCR protocols (not shown). We believe there are several potential explanations. There could be mtDNA deletions that either do not encompass the *MT‐ND4* region or stretch over both *MT‐ND1* and *MT‐ND4* hence they are not detectable with our real‐time assay. Alternatively, COX‐deficient fibres may have a mixed genetic aetiology and in some of them the observed respiratory defect may be unrelated to mtDNA. The analysis of homogenate DNA did not show a significant level of mtDNA deletion in any of the patients. Two patients had approximately 20% of mtDNA heteroplasmy; however, this is below the sensitivity of this assay [15, 16].

An interesting trend was observed in the level of mtDNA deletion (% mtDNA heteroplasmy) in four groups of previously characterized fibres: COX‐deficient, COX‐intermediate, complex I‐deficient and COX‐normal. As expected, the highest mutation load was found in COX‐deficient fibres with median of 23.3%, then gradually lower in the remaining three groups (median of 9.4, 1.6 and 0 for intermediate, complex I deficient and COX‐normal groups respectively). These differences, however, reached statistical significance only for COX‐normal *vs.* COX‐deficient cells (Mann–Whitney test, *P* = 0.0003) and COX‐normal *vs.* COX‐intermediate (*P* = 0.0135). The test performed was corrected for multiple testing and acceptable statistical confidence level was brought down to 2.5% (*P* of 0.025). There were cells, in all groups, which did not contain any detectable mtDNA deletions (Figure 5 **c**).

### Inflammatory infiltrate correlates with number of respiratory‐deficient fibres in sIBM muscle

**Figure 6:**
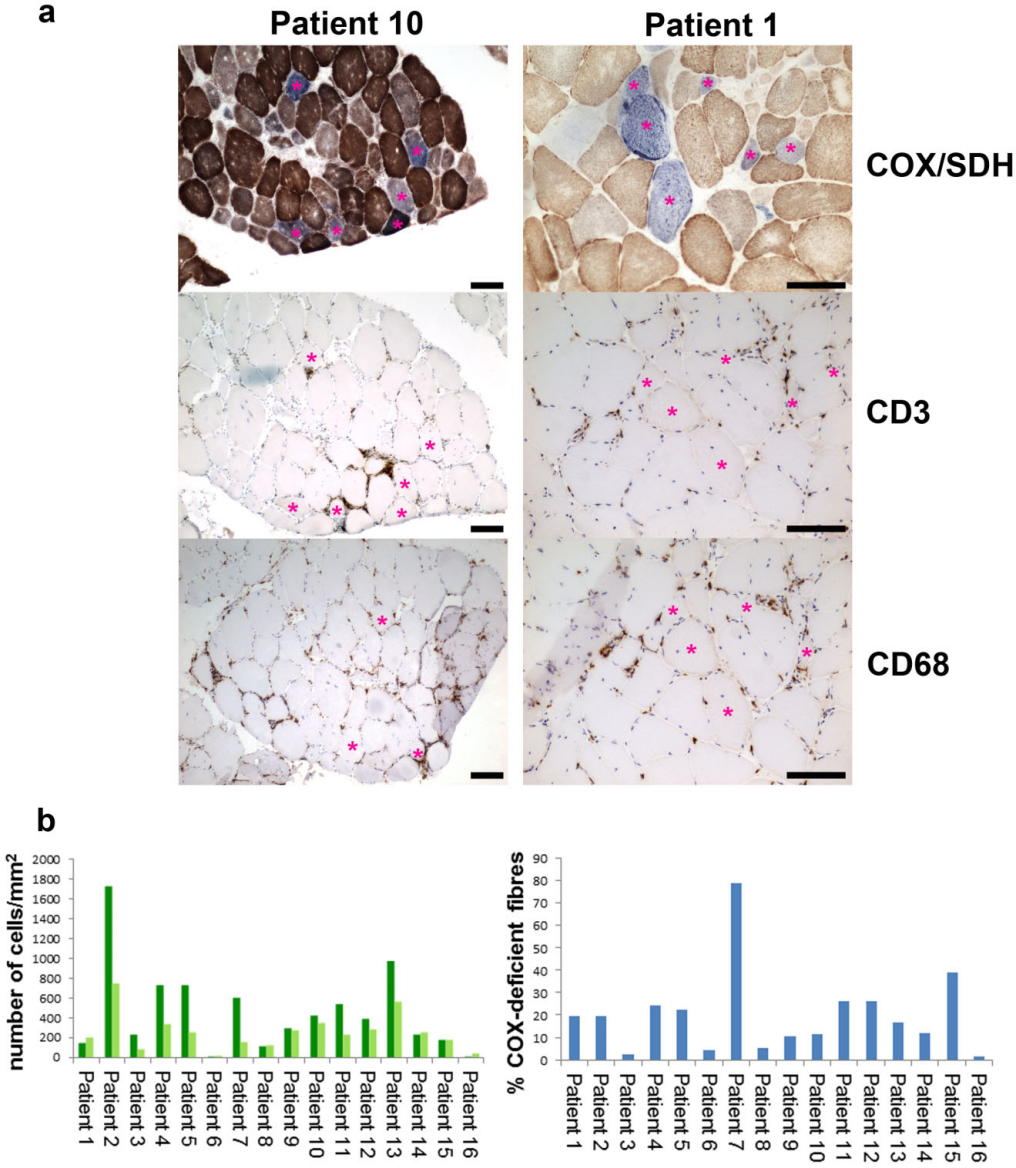
Correlation between severity of immune infiltration and number of respiratory‐deficient fibres in sIBM tissue. (**a**) Serial muscle sections from two patients showing COX/SDH combined histochemistry, T lymphocytes (CD3) and macrophages (CD68). Pink asterisks depict COX‐deficient and COX‐intermediate fibres throughout the sections; only some appear to be spatially associated with immune cells. (**b**) Graphs showing numbers of T cells (dark green bars) and macrophages (light green bars) counted in a representative area of each biopsy. There is a correlation between the number of invading CD3‐positive cells and COX‐deficient muscle fibres with the *P* of 0.007 and, a less strong correlation, between CD68‐positive cells and COX‐deficient fibres with *P* of 0.04 (fitted line plot). The scale bars measure 100 μm.

We investigated the inflammatory status of the sIBM muscle biopsies in relation to the levels of respiratory deficiency using immunohistochemical labelling for two antigens: CD3 and CD68 in order to identify populations of T lymphocytes and macrophages respectively (Figure 6 **a**). Consistent with findings from other groups, the CD3‐positive population was more abundantly represented than the CD68‐positive group [20]. We found a significant relationship between the number of fibres with mitochondrial abnormalities and immune cells residing within the tissue (Figure 6 **b**); stronger for CD3‐positive T cells (*r* = 0.66, *P* = 0.007) than macrophages (*r* = 0.24, *P* = 0.04). Although spatial association between immune cells and respiratory‐deficient fibres was only occasionally observed in the tissue, positive correlation between the total amount of inflammation and the degree of mitochondrial damage would strongly suggest a causative link between them (Figure 6).

### Relationship between COX deficiency and atrophy in sIBM muscle

**Figure 7:**
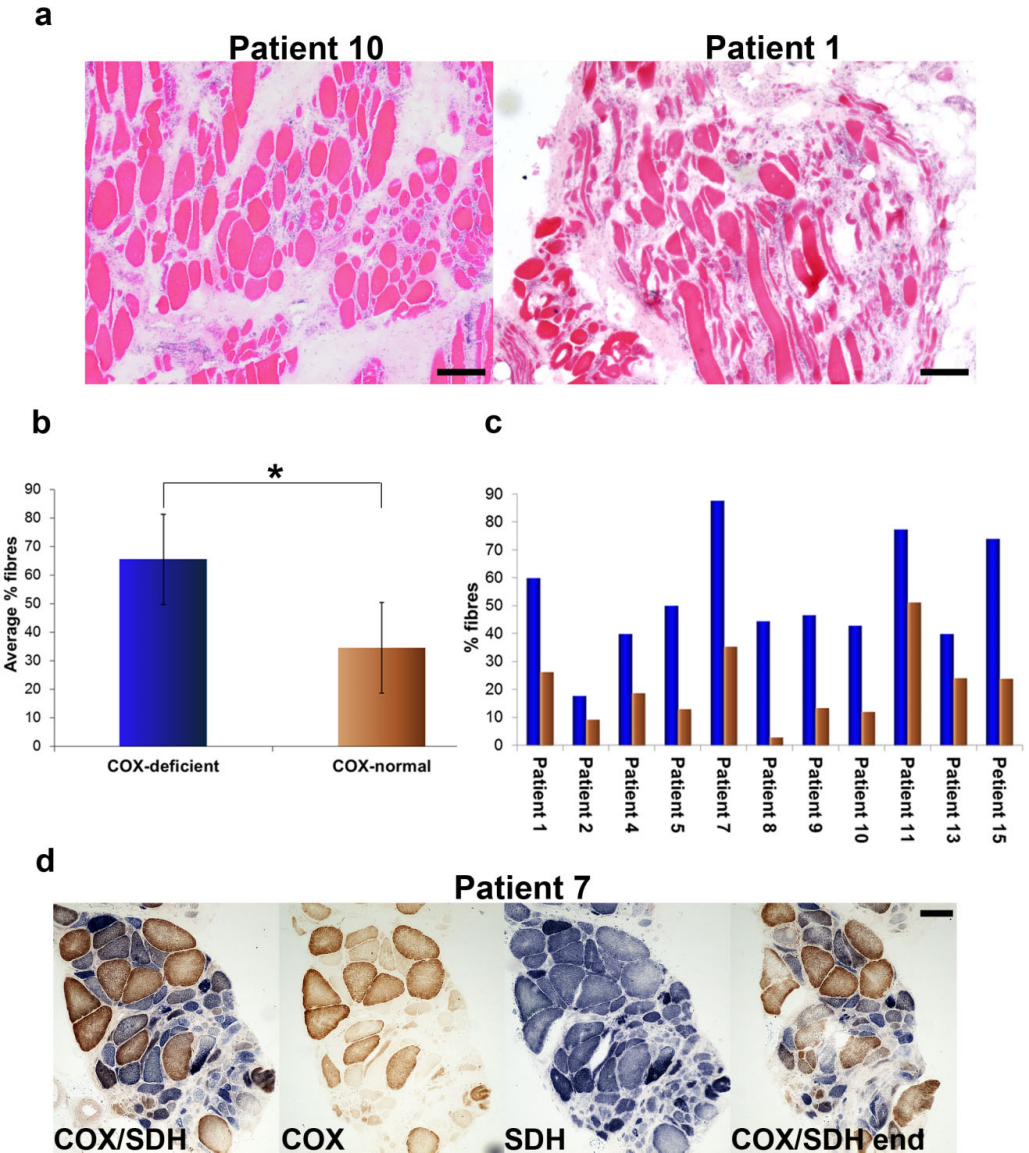
Atrophy in sIBM muscle sections. (**a**) H&E stained biopsy sections from two patients showing great variety in size of individual muscle fibres. (**b**) Average percentages of atrophic COX‐normal and COX‐deficient in the whole atrophic population measured in 11 cases. Statistically significant difference was reached between COX‐deficient and COX‐normal groups (*P = 0.009, one‐sample *T*‐test). (**c**) Percentages of atrophic cells in COX‐deficient and COX‐normal populations in 11 individual patients. The difference between contribution of COX‐deficient atrophic cells and COX‐normal counterparts in the overall COX‐deficient and COX‐normal groups respectively was highly significant (*P* < 0.0001, paired *T*‐test). (**d**) COX/SDH histochemistry carried out on a biopsy from patient 7, which demonstrates high contribution of atrophic respiratory‐deficient fibres to the entire COX‐deficient cell population (‘COX/SDH’ signifies first section in the series and ‘COX/SDH end’ the last section). The scale bars measure 100 μm.

A cardinal clinical feature of sIBM is muscle wasting, a consequence of degeneration of muscle fibres, and therefore we investigated atrophy in the sIBM biopsies (Figure 7 **a**). According to commonly accepted criteria atrophic fibres were those which smallest diameter measured less than 30 μm [21]. Analysis of average percentage of COX‐deficient and COX‐normal atrophic fibres in the entire atrophic population clearly shows that COX‐deficient fibres dominate over COX‐normal fibres (one‐sample *T*‐test, *P* = 0.009) (Figure 7 **b**). We also compared the proportion of atrophic fibres in the COX‐deficient and COX‐normal populations in individual sIBM cases (10 cases). We found that in all of the biopsies examined, the percentage of COX‐deficient atrophic fibres in the total COX‐deficient population was markedly higher than that of COX‐normal atrophic fibres in the overall COX‐normal population (paired *T*‐test, *P* < 0.0001). This would suggest that COX‐deficient cells are more prone to degeneration than COX‐normal fibres (Figure 7 **c**). Images of COX and SDH activity in a representative biopsy from patient 7 show the degree of atrophy in the respiratory normal and deficient subpopulations of muscle cells (Figure 7 **d**).

## Discussion

Mitochondrial dysfunction is common and often very prominent in muscle from sIBM patients. We demonstrate that there are abnormalities in the expression of individual mitochondrial respiratory complexes, and propose that a protocol combining mitochondrial respiratory subunit expression and COX/SDH activity provides a clearer picture of the mitochondrial changes in sIBM. We have explored the nature of the mitochondrial defect in individual muscle fibres and, in accordance with previous reports, we show that some of them accumulate high level of clonally expanded mtDNA deletions. We also show that respiratory‐deficient fibres are more prone to atrophy indicating that the mitochondrial defect has a direct effect on muscle wasting. In addition, there is a clear correlation between the overall percentage of respiratory‐deficient fibres and the degree of T lymphocyte infiltrate. Thus there seems to be a direct link between the inflammation present in muscle environment and the mitochondrial defect.

Combining COX/SDH histochemistry with immunohistochemical analysis of protein levels of selected mitochondrial respiratory chain components, we characterized three distinct groups of cells with mitochondrial abnormalities. Fibres with isolated complex I deficiency present the least severe phenotype. COX‐intermediate fibres show reduced COX activity with relatively preserved COX‐I protein, but loss of complex I. Finally, COX‐deficient cells have lost complex I and IV protein expression as well as COX enzymatic activity. This suggests a progressive nature of mitochondrial dysfunction which begins with complex I downregulation. We believe that by determining protein levels of complex I in sIBM muscle biopsies we are able to detect mitochondrial changes at the earliest stage of the disease. Therefore we postulate that the loss of complex I protein subunits, rather than COX activity, serves as a more sensitive marker of mtDNA damage in muscle from patients with sIBM. In our cohort of 16 patients we did not see a clear correlation between the number of COX‐deficient or complex I‐deficient and severity of disease progression. This finding does not dismiss a possibility that such correlation would be observed in a larger study. We believe that a combined approach of COX/SDH histochemistry and respiratory chain immunohistochemistry is valuable in understanding basic biology of mtDNA damage accumulation and its consequence on the muscle, although as yet its use in clinical practice is uncertain.

Consistent with a previous report from Oldfors *et al*. we found clonally expanded mtDNA deletions in COX‐deficient fibres [6]. Significant increase in a proportion of respiratory‐deficient muscle fibres, above the level predicted by normal ageing, is unique to sIBM and not observed in other inflammatory myopathies including polymyositis and dermatomyositis [22]. Interestingly, not all of the respiratory‐deficient single fibres had detectable level of deletion using our real‐time PCR assay. This finding is entirely compatible with results previously obtained by our laboratory studying patients with multiple mtDNA deletions due to nuclear gene disorders affecting mtDNA maintenance and in normal ageing [15, 16]. It is possible that deletions in these fibres remove a fragment of mtDNA which does not contain *MT‐ND4* region and thus they are not detected by the real‐time PCR assay we performed. The assay will also not detect a deletion which encompasses both *MT‐ND4* and *MT‐ND1*. A detailed analysis of mtDNA copy number excluded mtDNA depletion as the cause of respiratory deficiency in these cells. Finally, it is also possible that a clonally expanded point mutation is causing respiratory deficiency; a tRNA point mutation present at a very high level in a muscle fibre from a sIBM patient was previously reported [23]. However, this does not explain all the findings in our patients as sequencing analysis of the entire mitochondrial genome, from five single COX‐deficient cells, failed to detect any pathogenic point mutations.

We also investigated immune infiltrate and its potential relationship with respiratory defect in muscle fibres. Interestingly, a strong correlation exists between the severity of immune cell invasion and the number of fibres with mitochondrial abnormalities. We assessed the size of CD3‐positive population, which is an overall pool of T lymphocytes combining all subtypes including cytotoxic, helper, memory and regulatory T cells. A number of studies describe cytotoxic CD8‐positive T cells as a dominant group of lymphocytes in sIBM muscle tissue. FOXP3‐positive regulatory T cells have recently been reported to reside in muscles from inflammatory myopathies including sIBM and, interestingly, their number is proportional to the number of cytotoxic T cells [24]. In order for the mtDNA mutation to accumulate to the level of detection the muscle fibre must be spared from T cell mediated lysis which can only be granted by regulatory T cells. Scarcity of fibre necrosis as opposed to fibre atrophy in sIBM is supportive of this notion. It is intriguing why mtDNA damage has not been reported in other inflammatory myopathies. COX‐deficient fibres are occasionally found in dermatomyositis; however, these are believed to arise as a consequence of ischemia, based on their location associated with blood vessels [25]. We speculate that the long‐term inflammatory environment in sIBM must be important. It is worth pointing out that although we rarely saw a colocalization of immune cells with respiratory‐deficient muscle fibres in sIBM sections, it does not exclude a potential immune‐mediated mtDNA damage. The mtDNA rearrangements we identified in COX‐deficient fibres had clonally expanded over a period of many years and the inflammatory cells responsible for the initial insult had time to move away from the area [26]. Therefore the degree of inflammation likely represents the amount of damage inflicted overall which is then followed by the clonal expansion event. It is also worth noting that an increase in COX‐deficient muscle fibres has been reported in patients with multiple sclerosis, another chronic inflammatory condition [10].

A question remains as to whether accumulation of mtDNA mutation has any phenotypic effect on muscle fibres. This study has shown that respiratory‐deficient fibres were more prone to atrophy than fibres with normal respiration. This observation is strongly supported by data from studies investigating the effects of respiratory deficiency in ageing muscle [27, 28], although, in our experience as a diagnostic centre, we do not see a significant amount of atrophy in respiratory‐deficient fibre pool in patients with primary mtDNA mutations. If atrophy is indeed caused by mitochondrial deficiency in sIBM, and it is an irreversible process ultimately leading from gradual size reduction to degeneration of a fibre, then the contribution of mitochondrial involvement to the muscle wasting is important.

This study adds new information to the role of mitochondria in sIBM but many challenges remain. Our study was necessarily limited by the number of patients available, the lack of longitudinal data, counting based on a limited number of fibres and the patchy nature of the inflammatory involvement in inclusion body myositis meaning we could only study representative areas. Perhaps the most important challenge remains is mechanism by which the inflammation leads to the mitochondrial defect. We hypothesize a link involving cross‐talk between inflammation, mtDNA rearrangement accumulation and atrophy in sIBM (Figure S1). Once established these defects will inevitably progress, which may explain the lack of clinical benefit from immunosuppression in patients who typically present with a prolong duration of their disease. Whether very early intervention would limit the mitochondrial involvement is not known. Finally, we see remarkable variation in the degree of mitochondrial involvement between patients and we are uncertain whether this reflects different pathogenic mechanisms or the sensitivity of the mitochondrial genome to mutate in some patients. A greater understanding of these disease mechanisms may lead to new therapeutic avenues for patients with this progressive muscle disease.

## Funding

This work was funded by Newcastle University Centre for Brain Ageing and Vitality [supported by the Biotechnology and Biological Sciences Research Council, Engineering and Physical Sciences Research Council, Economic and Social Research Council and Medical Research Council (G0700718)], the UK NIHR Biomedical Research Centre in Age and Age Related Diseases award to the Newcastle upon Tyne Hospitals NHS Foundation Trust, MRC Centre for Neuromuscular Disease (G000608‐1), The Wellcome Trust Centre for Mitochondrial Research (096919/Z/11/Z), The Lily Foundation and the UK NHS Highly Specialised ‘Rare Mitochondrial Disorders of Adults and Children’ Service.

## Disclosure statement

The authors disclose no conflicts of interest including any financial, personal or other relationships with other people or organizations which could inappropriately influence their work.

## Author contributions

DMT designed the study. KAR carried out the experimental work. JM provided the tissue and carried out the clinical evaluation of the samples. JPG performed statistical analyses. MCR verified quantitative analysis of muscle biopsies. RWT helped with interpretation of the data. KAR wrote the manuscript which was revised by DMT, RWT, JM and JPG.

## Supporting information


**Figure S1.** We hypothesize that the long‐term inflammation in sIBM is a key trigger to the mtDNA damage, leading to the accumulation of clonally expanded mtDNA deletions. Mitochondrial DNA mutations may form as a result of inflammatory insult such as RONS (reactive oxygen and nitrogen species) or inflammatory cytokines present in the environment. Mitochondrial mutations accumulated above a critical threshold can cause a phenotypic effect such as muscle atrophy, splitting and breakage (Bua *et al*. 2002), explaining the correlation of respiratory deficiency with muscle atrophy. As sIBM is considered an autoimmune disease we may speculate that an initial viral infection triggers breakage of immune tolerance mediated by CD8‐positive T cells and causes an autoimmune response. Secretion of inflammatory cytokines results in upregulation of MHC I and the co‐stimulatory molecules by muscle fibres, which then become a target for an immune attack. The inflammatory environment may directly cause double‐strand breaks in mtDNA (dsDNA breaks). Alternatively, MHC I upregulation may cause ER stress response which leads to accumulation of misfolded toxic proteins such as β‐amyloid. The latter augments inflammatory reaction and associates with mitochondrial membranes disrupting intramitochondrial processes. Faulty repair mechanisms subsequently lead to the accumulation of damaged mtDNA molecules in the cell causing respiratory deficiency (Krishnan *et al*. 2008).
**Table S1.** Clinical data of cases analysed in the study.
**Table S2.** Description and source of the antibodies used for IHC assessments.
