# Supplementary material for: Mitochondrial and inflammatory changes in sporadic inclusion body myositis
Source: Neuropathol Appl Neurobiol. 2015 Mar 4;41(3):288–303. doi: 10.1111/nan.12149 (PMC4833191; doi:10.1111/nan.12149)
Supplement: Supplementary file 1 — Figure S1. We hypothesize that the long‐term inflammation in sIBM is a key trigger to the mtDNA damage, leading to the accumulation of clonally expanded mtDNA deletions. Mitochondrial DNA mutations may form as a result of inflammatory insult such as RONS (reactive oxygen and nitrogen species) or inflammatory cytokines present in the environment. Mitochondrial mutations accumulated above a critical threshold can cause a phenotypic effect such as muscle atrophy, splitting and breakage (Bua et al. 2002), explaining the correlation of respiratory deficiency with muscle atrophy. As sIBM is considered an autoimmune disease we may speculate that an initial viral infection triggers breakage of immune tolerance mediated by CD8‐positive T cells and causes an autoimmune response. Secretion of inflammatory cytokines results in upregulation of MHC I and the co‐stimulatory molecules by muscle fibres, which then become a target for an immune attack. The inflammatory environment may directly cause double‐strand breaks in mtDNA (dsDNA breaks). Alternatively, MHC I upregulation may cause ER stress response which leads to accumulation of misfolded toxic proteins such as β‐amyloid. The latter augments inflammatory reaction and associates with mitochondrial membranes disrupting intramitochondrial processes. Faulty repair mechanisms subsequently lead to the accumulation of damaged mtDNA molecules in the cell causing respiratory deficiency (Krishnan et al. 2008). Table S1. Clinical data of cases analysed in the study. Table S2. Description and source of the antibodies used for IHC assessments. [file NAN-41-288-s001.zip › nan_12149-sup-0001-figureS1.pptx]

## Slide 1
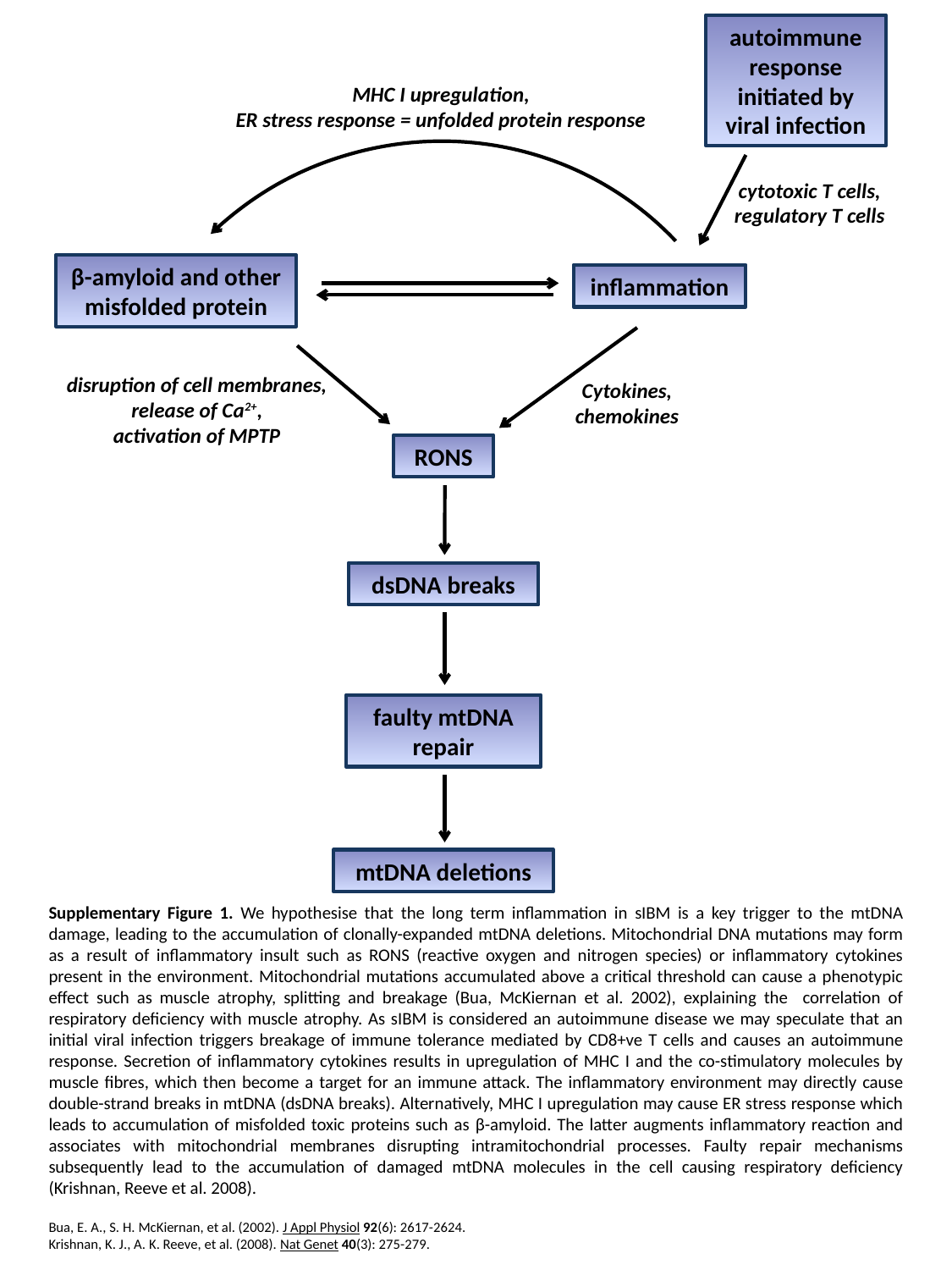

autoimmune response initiated by viral infection
MHC I upregulation,
ER stress response = unfolded protein response
cytotoxic T cells,
regulatory T cells
β-amyloid and other misfolded protein
inflammation
disruption of cell membranes,
release of Ca2+,
activation of MPTP
Cytokines,
chemokines
RONS
dsDNA breaks
faulty mtDNA repair
mtDNA deletions
Supplementary Figure 1. We hypothesise that the long term inflammation in sIBM is a key trigger to the mtDNA damage, leading to the accumulation of clonally-expanded mtDNA deletions. Mitochondrial DNA mutations may form as a result of inflammatory insult such as RONS (reactive oxygen and nitrogen species) or inflammatory cytokines present in the environment. Mitochondrial mutations accumulated above a critical threshold can cause a phenotypic effect such as muscle atrophy, splitting and breakage (Bua, McKiernan et al. 2002), explaining the correlation of respiratory deficiency with muscle atrophy. As sIBM is considered an autoimmune disease we may speculate that an initial viral infection triggers breakage of immune tolerance mediated by CD8+ve T cells and causes an autoimmune response. Secretion of inflammatory cytokines results in upregulation of MHC I and the co-stimulatory molecules by muscle fibres, which then become a target for an immune attack. The inflammatory environment may directly cause double-strand breaks in mtDNA (dsDNA breaks). Alternatively, MHC I upregulation may cause ER stress response which leads to accumulation of misfolded toxic proteins such as β-amyloid. The latter augments inflammatory reaction and associates with mitochondrial membranes disrupting intramitochondrial processes. Faulty repair mechanisms subsequently lead to the accumulation of damaged mtDNA molecules in the cell causing respiratory deficiency (Krishnan, Reeve et al. 2008).
Bua, E. A., S. H. McKiernan, et al. (2002). J Appl Physiol 92(6): 2617-2624.
Krishnan, K. J., A. K. Reeve, et al. (2008). Nat Genet 40(3): 275-279.
